# ‘We agreed that they could conduct an autopsy on my brother’

**DOI:** 10.1098/rstb.2008.4023

**Published:** 2008-11-27

**Authors:** Komit Poki

**Affiliations:** Yagareba Village, c/o Papua New Guinea Institute of Medical ResearchPO Box 60, Goroka, EHP 441, Papua New Guinea

I was only a child when Dr Carleton Gajdusek, Michael Alpers and Shirley Lindenbaum conducted research on kuru in the South Fore. They patrolled around the villages and saw how many of our people were dying of kuru and the number of orphans in our villages.

My late brother, Mr Animo Kaguya, was the only member of our family involved in the early research on kuru; he assisted in carrying equipment, transporting specimens and locating kuru patients. He was living happily with his family when he started to show signs of kuru in 2002; later, Michael came and examined him and confirmed that he did indeed have kuru.

Jerome Whitfield and his colleagues from the Papua New Guinea Institute of Medical Research approached our family and we agreed that they could conduct an autopsy on my brother. The autopsy was performed well in the village and the Kuru Project then assisted with the funeral preparations.

Today, it looks as though kuru has nearly disappeared, but other common diseases such as malaria, TB, typhoid, asthma and AIDS are claiming innocent lives in Okapa District. If an opportunity arises for medical scientists to work on other diseases in our district, we would be very happy.

In some of the villages where medical scientists once worked, kuru research is still being conducted and those of us who understand the importance of the work are happy to collaborate to help future generations of Papua New Guineans.

In conclusion, I thank the early kuru researchers for their work and for helping us to change our cultural practices that were harmful.

